# Mediation Analysis With Intermediate Confounding: Structural Equation Modeling Viewed Through the Causal Inference Lens

**DOI:** 10.1093/aje/kwu239

**Published:** 2014-12-11

**Authors:** Bianca L. De Stavola, Rhian M. Daniel, George B. Ploubidis, Nadia Micali

**Keywords:** eating disorders, estimation by combination, G-computation, parametric identification, path analysis, sensitivity analysis

## Abstract

The study of mediation has a long tradition in the social sciences and a relatively more recent one in epidemiology. The first school is linked to path analysis and structural equation models (SEMs), while the second is related mostly to methods developed within the potential outcomes approach to causal inference. By giving model-free definitions of direct and indirect effects and clear assumptions for their identification, the latter school has formalized notions intuitively developed in the former and has greatly increased the flexibility of the models involved. However, through its predominant focus on nonparametric identification, the causal inference approach to effect decomposition via natural effects is limited to settings that exclude intermediate confounders. Such confounders are naturally dealt with (albeit with the caveats of informality and modeling inflexibility) in the SEM framework. Therefore, it seems pertinent to revisit SEMs with intermediate confounders, armed with the formal definitions and (parametric) identification assumptions from causal inference. Here we investigate: 1) how identification assumptions affect the specification of SEMs, 2) whether the more restrictive SEM assumptions can be relaxed, and 3) whether existing sensitivity analyses can be extended to this setting. Data from the Avon Longitudinal Study of Parents and Children (1990–2005) are used for illustration.

The epidemiologic literature on causal inference is alight with contributions dedicated to the study of mediation. (A PubMed search for articles on mediation analysis in epidemiology produced 118 “hits” for articles published in 2012 and 110 “hits” for articles published in 2013.) The topic owes its origins, however, to an older body of literature that is well known in the social sciences. This school is often referred to as the “Baron and Kenny approach” (1, 2) but is linked to Sewall Wright's path analysis (3) and its extension, structural equation models (SEMs) (4). It includes several important publications that are less well known in the epidemiologic literature (5–10).

Contributions from the causal inference school have formalized and generalized notions intuitively developed in the SEM school, first by defining (using potential outcomes) precisely what is meant by direct and indirect effects, then by giving clear assumptions under which they can be identified, and lastly by generalizing the statistical methods available for carrying out such analyses to allow for nonlinearities, interactions, discrete outcomes, and semiparametric estimation (11–26).

With a few notable exceptions (11, 27–29), the literature on natural direct and indirect effects focuses predominantly on *nonparametric identification*, which leads to the strong assumption of “no intermediate confounders”—that is, that no confounders (measured or unmeasured) of the mediator and outcome may be affected by the exposure. By relying on parametric models, however, such confounders are naturally dealt with in the SEM framework. Therefore, it is pertinent and timely to revisit SEMs with intermediate confounders, armed with the formal definitions and (parametric) identification assumptions from causal inference to reconcile the 2 approaches in this particular context.

In this article, we review how paths are traced in order to derive direct and indirect effects in simple linear SEMs which include intermediate confounders but exclude nonlinearities, and show their equivalence to the definitions based on potential outcomes. We then investigate how different parametric assumptions for identification of the natural effects in the presence of intermediate confounders affect the specification of an extended SEM that includes nonlinearities. We further investigate whether the usual SEM assumption of “no omitted influences” of any pair of variables in the system can be relaxed when estimation of the natural effects is the goal. Finally, we widen existing sensitivity analyses to the setting with intermediate confounding, exploiting the SEM framework.

## THE 2 FRAMEWORKS

### Settings and aims

**Figure 1.**
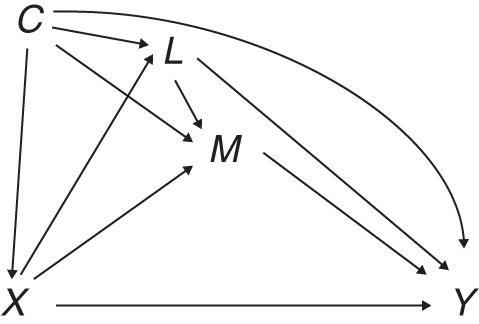
Causal diagram for exposure *X*, mediator *M*, outcome *Y*, background confounder *C*, and intermediate confounder *L*.

We will discuss settings involving an exposure *X*, an outcome *Y*, a mediator *M*, background confounders *C* of 1 or more of the relationships *X-Y*, *M-Y*, and *X-M*, and intermediate confounders *L* of the *M-Y* relationship (Figure 1). The aim is to separate the causal effect of *X* acting along pathways that include *M* from the causal effect of *X* acting along other pathways that do not involve *M* (the *indirect* and *direct* effects, respectively).

For simplicity, we let *X* be a binary variable and assume that observations are not affected by missingness or measurement error.

### The causal inference framework

The causal inference framework (11, 12) invokes *potential outcomes* (30). For mediation analysis, these are: *M*(*x*), the potential value of *M* if *X* had been set, possibly counter to fact, to the value *x*; *Y*(*x*, *m*), the potential value of *Y* if *X* had been set to *x* and *M* to *m*; and *Y*(*x*, *M*(*x*′)), the *composite* potential value of *Y* if *X* had been set to *x* and *M* to *M*(*x*′).

Several definitions of direct and indirect effects have been proposed, with the choice depending on the causal question being addressed. We focus here on those most widely used and define them as linear contrasts, although definitions on other scales have been given (31–33).

### Definitions

The controlled direct effect (CDE) of *X* on *Y* when *M* is controlled at *m*, CDE(*m*), and the pure natural direct effect (PNDE) of *X* on *Y* (11, 12) are

$$\begin{array}{rl} \text{ CDE}(m) & =E\{Y(1,m)\}-E\{Y(0,m)\}.\\ \text{PNDE} & =E\{Y(1,M(0))\}-E\{Y(0,M(0))\}. \end{array}$$

CDE(*m*) is a comparison of 2 hypothetical worlds where, in the first, *X* is set to 1 and, in the second, *X* is set to 0, while in both worlds *M* is set to *m*. The PNDE is also a comparison of 2 hypothetical worlds where *X* is set to 0 or 1 but *M* is set to take its *natural* value *M*(0). Because in each of these comparisons *M* is set at the same value in both worlds (at least within the individual), they are measures of effects of *X* unmediated by *M*, that is, “direct.”

The complement of the PNDE is the total natural indirect effect (TNIE) of *X* on *Y* (11, 34):

TNIE=TCE−PNDE=E{Y(1,M(1))}−E{Y(1,M(0))},

where TCE = *E*{*Y*(1)} − *E*{*Y*(0)} represents the total causal effect. The TNIE is a comparison of 2 hypothetical worlds in which *X* is set to 1 in both, while *M* changes from its *natural* value when *X* is 1 to its *natural* value when *X* is 0. Intuitively, this is an indirect effect, since it captures the part of the effect of *X* on *Y* that is transmitted by *M*. There is no equivalent complement of CDE(*m*) (35).

### Assumptions

#### In the absence of intermediate confounders

Identification of these estimands is possible if certain assumptions hold. Those most commonly invoked are specific versions of *no interference*, *consistency*, and *conditional exchangeability*.

Briefly, in the setting with no intermediate confounders and for CDE(*m*), the assumption of no interference states that an individual's outcome is not influenced by the exposure status of another person (36–39) and also that the mediator value for one individual has no effect on the outcome in another. The assumption of consistency states that *Y*(*x*, *m*) equals *Y* among subjects with observed exposure level *X* = *x* and mediator level *M* = *m* (40–43). The assumption of conditional exchangeability states that once individuals are stratified according to confounders *C*, their allocation to *X* is essentially “random” within these strata, and once they are stratified according to *X* and *C*, their allocation to *M* is essentially random within those strata. More formally, conditional exchangeability states that Y(x)⊥⊥X|C and Y(x,m)⊥⊥M|C,X, implying no *X*-*Y* confounding conditionally on *C* and no *M*-*Y* confounding conditionally on *C* and *X* (30, 44). Under these extended assumptions, CDE(*m*) is nonparametrically identified by regression standardization. For discrete *C* (45, 46),

(1) $$\;\begin{array}{rl} \mathrm{CDE}(m)= & \sum_{c}\{E(Y|X=1,M=m,C=c)\\ & -E(Y|X=0,M=m,C=c)\}\;\text{Pr}(C=c). \end{array}$$

The sums here are replaced by integrals and Pr(*C* = *c*) by the corresponding density, if *C* is continuous.

In order to identify the PNDE, the assumption of no interference is expanded also to mean that the exposure of one individual has no effect on the mediator of another; the assumption of consistency is expanded also to mean that *M*(*x*) = *M* when *X* = *x* and that *Y*(*x*, *M*(*x*)) = *Y* when *X* = *x* (denoted *generalized consistency* or *composition* (46)); and the assumption of conditional exchangeability is expanded to mean that there is also no *X*-*M* confounding conditional on *C* (formally, M(x)⊥⊥X|C).

Under these extended assumptions, and when *M* and *C* are discrete, the PNDE is nonparametrically identified (12, 45, 46) by

(2) $$\begin{array}{rl} & \sum_{c}\sum_{m}\{E(Y|X=1,M=m,C=c)\\ & \;\;-E(Y|X=0,M=m,C=c)\}\\ & \;\;\times \text{Pr}(M=m|X=0,C=c)\;\text{Pr}(C=c). \end{array}$$

The same assumptions are invoked to nonparametrically identify the TNIE, leading to (46)

(3) $$\begin{array}{rl} \text{TNIE} & =\sum_{c}\sum_{m}E(Y|X=1,M=m,C=c)\\ & \;\times \{\text{Pr}(M=m|X=1,C=c)\\ & \;-\text{Pr}(M=m|X=0,C=c)\}\;\text{Pr}(C=c). \end{array}$$

For continuous *C*/*M*, summations are replaced by integrals and probabilities by density functions (see part A of the Web Appendix, available at http://aje.oxfordjournals.org/). Equations 2 and 3 are known as the *mediation formula* (45).

#### In the presence of intermediate confounders

Identifying CDE(*m*) in the presence of intermediate confounders *L* can be achieved by adapting the assumption of no unaccounted *M*-*Y* confounding to include conditioning on *L* (Y(x,m)⊥⊥M|C,X,L) and updating identification formula 1 (equation 1) to include the contribution via *L*. This is commonly referred to as the *G-computation formula* (46, 47) (Web Appendix, part B).

In contrast, identification of the natural effects, PNDE and TNIE, additionally involves some parametric restrictions on the relationships among *X*, *M*, *L*, and *Y*. Originally the restriction was stated by Robins and Greenland (11) as no *X*-*M* interaction at an individual level. Alternatively, Petersen et al. (27) suggested assuming that, conditional on *C*, the CDE does not vary with *M*(0). Under either of these additional parametric assumptions, PNDE and TNIE are identified by formulae that are extensions of equations 2 and 3. (Identification can also be obtained under certain “no-3-way-interaction” assumptions when the exposure is randomly assigned (48) or under no average *L*-*M* interaction in a nonparametric SEM with mutually independent errors (29).)

### Estimation

Several approaches have been proposed for the estimation of these estimands, with standard errors typically obtained by sandwich estimation or bootstrapping (for a review, see Vansteelandt (46)). Among them, an extension of Robins' (47) G-computation that incorporates the mediation formula posits regression models for each of the (conditional) expectations/probabilities/densities in the identifying equations, estimates their parameters (e.g., using maximum likelihood), and then plugs these estimates into the sums/integrals above (47, 49). When the G-computation formula is too cumbersome to be evaluated analytically, the integration can be approximated through Monte Carlo simulation (47, 50) (see Appendix 2). The advantage of this approach is efficiency when all models are correctly specified, as well as flexibility. Essentially any combination of types (binary/categorical/continuous) of outcomes, mediators, and intermediate confounders can be modeled with little restriction on the assumed models, although the resulting complexities are a drawback (26).

To lessen the reliance on parametric modeling assumptions, many alternative semiparametric estimation approaches have been suggested, in particular G-estimation of structural nested models (21), inverse probability weighting of marginal structural models (20), doubly and multiply robust methods that combine 1 or more of these approaches (24, 25), and multiply robust methods based on targeted maximum likelihood (51).

### The SEM framework

**Figure 2.**
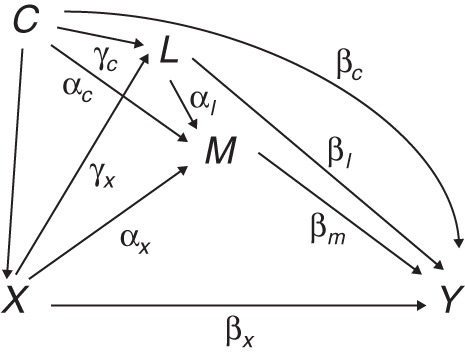
Structural equation model for exposure *X*, mediator *M*, outcome *Y*, background confounder *C*, and intermediate confounder *L* (error terms omitted for simplicity).

Unlike the above, the definitions of direct and indirect effects given in the SEM literature depend on the specification of a particular statistical model (49). In the setting of Figure 2 (with single *C* and *L*), the following model for continuous *Y*, *M*, and *L* could be specified:

(4) $$\left\{ \begin{array}{rl} L & =\gamma_{0}+\gamma_{x}X+\gamma_{c}C+\epsilon_{l}\\ M & =\alpha_{0}+\alpha_{x}X+\alpha_{l}L+\alpha_{c}C+\epsilon_{m}\\ Y & =\beta_{0}+\beta_{x}X+\beta_{m}M+\beta_{l}L+\beta_{c}C+\epsilon_{y}, \end{array}\right.$$

where *X* and *C* are *exogenous* variables (no equations are specified for them), *Y*, *M*, and *L* are *endogenous* variables, and $\epsilon_{l}$, $\epsilon_{m}$, and $\epsilon_{y}$ are mean-zero error terms, uncorrelated with each other and with the exogenous variables. This is a linear path model for the joint distribution of *Y*, *M*, and *L* (4, 52).

Sequentially replacing the expression for *L* into the equation for *M* and that for *M* into the equation for *Y*, we obtain the reduced form of model 4 (equation 4):

$$\begin{array}{rl} Y & =(\beta_{0}+\alpha_{0}\beta_{m}+\alpha_{l}\beta_{m}\gamma_{0}+\beta_{l}\gamma_{0})+(\beta_{x}+\alpha_{x}\beta_{m}+\alpha_{l}\beta_{m}\gamma_{x}+\beta_{l}\gamma_{x})X\\ & \;+(\beta_{c}+\alpha_{c}\beta_{m}+\beta_{l}\gamma_{c}+\alpha_{l}\beta_{m}\gamma_{c})C+(\beta_{m}\epsilon_{m}+\alpha_{l}\beta_{m}\epsilon_{l}+\beta_{l}\epsilon_{l}+\epsilon_{y}). \end{array}$$

Here (β*_x_* + α*_x_*β*_m_* + α*_l_*β*_m_*γ*_x_* + β*_l_*γ*_x_*) is taken to represent the *total causal effect* of *X* on *Y*. It can be partitioned into the direct (not mediated by *M*) and indirect (mediated) effects of *X* by *tracing the paths* in Figure 2 that make up the total effect (52). The indirect effect is found by multiplying the parameters along each of the (directed) paths from *X* to *Y* that include *M* and summing them; here, this is (α*_x_*β*_m_* + γ*_x_*α*_l_*β*_m_*). The direct effect is the sum of the remainder, (β*_x_* + γ*_x_*β*_l_*). This is a more general version of the *product of coefficients* method (2, 13, 53).

Tracing the paths is possible only when the models for the endogenous variables are linear and do not include any interactions or other nonlinearities, although generalizations to settings with binary outcomes (via logit or probit regression) have been suggested, with standardization of the estimated parameters used to deal with their differences in scale across models (54). Other approaches within the SEM framework (i.e., without relying on counterfactuals) have also been proposed for general link functions and for models with interactions and other nonlinearities (9, 10, 49, 55), but these are only approximate and do not explicitly deal with settings with intermediate confounding.

### Assumptions and estimation

Depending on the author, the identifying assumptions given in the SEM literature vary in detail, but essentially they are (5, 7, 8, 52, 56):

1. Correct temporal order between *X*, *L*, *M*, and *Y*.
2. “No omitted influences” (8), or “no lack of self-containment” (7), or “no other hidden relevant causes” (52).
3. Correct functional forms of each equation in the model.
4. Accurate measurements of all of the observed variables.
5. Error terms that are uncorrelated with each other and with the exogenous variables.

The first 2 assumptions are structural, that is, causal, meaning that the regression equations fully reflect the underlying data-generating process and that they justify the apportioning of the mediation effects described above (7, 8, 52). For settings with intermediate confounders, “no omitted influences” is a stronger assumption than the conditional exchangeability assumption invoked in the causal inference literature, since it also involves no *L*-*Y* confounding.

The last 3 assumptions are statistical. The first refers to the linearity and additivity of the relationships among the variables, the second to the reliability of the available data, and the third to the behavior of the error terms. Requiring the error terms to be uncorrelated with each other and with the exogenous variables guarantees unbiased estimation of the model's parameters via least squares. These estimated parameters can then be combined to obtain estimates of the direct and indirect effects, with measures of their precision obtained via the delta method (6) or bootstrapping (57). Importantly, departures from the statistical assumptions have repercussions for the structural ones. Correlated error terms—or correlated error terms and exogenous variables—would indicate departures from the structural assumption of no omitted relevant variables (52). Departures from the assumption of accurate measurements of the observed variables would lead to biased estimates of the model parameters and consequently of the mediation parameters (58).

Interestingly, the SEM literature does not mention the assumptions of no interference and consistency invoked by the causal inference literature, even though both are required for the estimated parameters to be interpreted as causal (59).

## INSIGHTS

The causal inference estimands are defined in generality, although identification is achieved only parametrically when intermediate confounding is present. The SEM estimands are derived from specific parametric structural models that naturally include intermediate confounders. The 2 approaches are therefore very different, but they converge under certain scenarios. We believe that understanding their overlap when intermediate confounding is present can offer useful analytical insights.

### Equivalence in estimands

The SEM approach to mediation applied to model 4 identifies the mediated effect of *X* on *Y* via *M* as (α*_x_*β*_m_* + γ*_x_*α*_l_*β*_m_*) and the nonmediated one as (β*_x_* + γ*_x_*β*_l_*).

Under the same structural and parametric assumptions, the causal inference estimands can be written in closed form (see Web Appendix, part B):

$$\;\begin{array}{rl} \mathrm{PNDE} & =\int_{c}\{\int_{l^{\;\prime }}\int_{m}\int_{l}\{E(Y|X=1,M=m,L=l,C=c)\;f_{L}(l|X=1,C=c)\\ & \;\;\;-E(Y|X=0,M=m,L=l,C=c)\;f_{L}(l|X=0,C=c)\}\;dl\\ & \;\;\;\times \;f_{M}(m|L=l^{\;\prime },X=0,C=c)\;f_{L}(l^{\;\prime }\;|\;X=0,C=c)\;dm\;dl^{\;\prime }\}\;f_{C}(c)\;dc\\ & =\int_{c}\{\int_{l^{\;\prime }}\int_{m}(\beta_{x}+\beta_{l}\gamma_{x})\;f_{M}(m|L=l^{\;\prime },X=0,C=c)\;f_{l}(l^{\;\prime }|X=0,C=c)\;dm\;dl^{\;\prime }\}\;f_{C}(c)\;dc\\ & =\beta_{x}+\beta_{l}\gamma_{x}. \end{array}$$

$$\;\begin{array}{rl} \text{CDE}(m) & =\int_{c}\{\int_{l}E\{(Y|X=1,M=m,C=c,L=l)\;f_{L}(l|X=1,C=c)\;dl\\ & \;\;\;-\int_{l}E(Y|X=0,M=m,C=c,L=l)\;f_{L}(l|X=0,C=c)\;dl\}\;f_{C}(c)\;dc\\ & =\int_{c}(\beta_{x}+\beta_{l}\gamma_{x})\;f_{C}(c)\;dc\\ & =\beta_{x}+\beta_{l}\gamma_{x}. \end{array}$$

$$\;\begin{array}{rl} \mathrm{TNIE} & =\int_{c}\{\int_{l^{\;\prime }}\int_{m}\int_{l}E(Y|X=1,M=m,L=l,C=c)\;f_{L}(l|X=1,C=c)\\ & \;\;\;\;\times \{\;f_{M}(m|X=1,L=l^{\;\prime },C=c)\;f_{L}(l^{\;\prime }|X=1,C=c)\\ & \;\;\;-\;f_{M}(m|X=0,L=l^{\;\prime },C=c)\;f_{L}(l^{\;\prime }|X=0,C=c)\}\;dl\;dm\;dl^{\;\prime }\}\;f_{C}(c)\;dc\\ & =\int_{c}\{\beta_{m}(\alpha_{x}+\alpha_{l}\gamma_{x})\}\;f_{C}(c)\;dc\\ & =\beta_{m}(\alpha_{x}+\alpha_{l}\gamma_{x}). \end{array}$$

Hence the estimands from the 2 approaches coincide when the same parametric assumptions are made; likewise in the simple setting without intermediate confounders (10, 13, 45, 49). Although these equivalences apply only to linear SEMs that have no interactions or other nonlinear terms involving *X*, *M*, and *L*, closed-form solutions for the causal estimands above are not restricted to these simple models. Appendix 1 shows the closed-form solutions obtained for a more general linear SEM:

(5) $$\left\{\begin{array}{ll} L & =\gamma_{0}+\gamma_{x}X+\gamma_{c}C+\epsilon_{l}\\ M & =\alpha_{0}+\alpha_{x}X+\alpha_{l}L+\alpha_{c}C+\alpha_{xl}XL+\epsilon_{m}\\ Y & =\beta_{0}+\beta_{x}X+\beta_{l}L+\beta_{ll}L^{2}+\beta_{m}M+\beta_{mm}M^{2}\\ & +\;\beta_{c}C+\beta_{xl}XL+\beta_{xm}XM+\epsilon_{y}, \end{array}\right.$$

where the residual terms are uncorrelated with each other and the explanatory variables in their equations and have constant variances $\sigma_{l}^{2}$, $\sigma_{m}^{2}$, and $\sigma_{y}^{2}$, respectively.

Parametric G-computation of the causal estimands above can then be achieved by combining the relevant estimated parameters of the assumed SEM, leading to what we refer to as *estimation by combination* (see Appendix 2 for its implementation in Mplus (Muthén and Muthén, Los Angeles, California); this implementation is more general than those in the papers by Valeri and VanderWeele (15) and Emsley et al. (60), which deal only with settings without *L*). Comparing the results obtained from analytical (i.e., by-combination) and Monte Carlo G-computation allows evaluation of the extent of the Monte Carlo error, as illustrated in the example.

### Understanding the assumptions required for parametric identification

Identifiability of the natural direct and indirect effects in the presence of intermediate confounding involves some parametric restrictions on the relationships among *X*, *M*, *L*, and *Y*. Specifically, Robins and Greenland (11) proposed the assumption of no individual *X*-*M* interaction—formally, that Y(1,m)−Y(0,m) is the same for all *m*. For settings in which parametric models for *Y*, *M*, and *L* are specified via linear regression, this can be formally examined.

For example, consider model 5 (equation 5). Assuming it is correctly specified, we see that

$$\begin{array}{rl} Y(1,m)-Y(0,m) & =\beta_{x}+\beta_{l}(L(1)-L(0))+\beta_{ll}(L(1{)}^{2}-L(0{)}^{2})\\ & \;+\beta_{xl}L(1)+\beta_{xm}m\\ & =\beta_{x}+\beta_{l}\gamma_{x}+\beta_{ll}\{\gamma_{x}^{2}+2\gamma_{x}(\gamma_{0}+\gamma_{c}C+\epsilon_{l})\}\\ & \;+\beta_{xl}(\gamma_{0}+\gamma_{x}+\gamma_{c}C+\epsilon_{l})+\beta_{xm}m, \end{array}$$

and thus the Robins and Greenland assumption holds if and only if $\beta_{xm}=0.$ Note that, had our model for *Y* included a term in *LM*, the Robins and Greenland assumption would also have constrained its coefficient (β*_lm_*) to be zero (in line with the constraint proposed by Tchetgen Tchetgen and VanderWeele (29)).

Petersen et al. (27) propose the alternative identifying assumption that, within levels of *C*, the CDE does not vary with *M*(0). Formally,

$$\begin{array}{rl} & E\{Y(1,m)-Y(0,m)|M(0)=m,C=c\}\\ & \;=E\{Y(1,m)-Y(0,m)|C=c\}. \end{array}$$

Again, assuming that model 5 is correct, we see that

$$\begin{array}{rl} M(0) & =\alpha_{x}+\alpha_{l}L(0)+\alpha_{c}C+\epsilon_{m}\\ & =\alpha_{x}+\alpha_{l}(\gamma_{0}+\gamma_{c}C+\epsilon_{l})+\alpha_{c}C+\epsilon_{m}. \end{array}$$

Conditional on *C*, therefore, we see that both *Y*(1, *m*) − *Y*(0, *m*) and *M*(0) are functions of $\epsilon_{l}$, except when $\beta_{ll}=\beta_{xl}=0.$ Note that, given our model, assuming that γ*_x_* = 0 (in place of β*_ll_*) or that α*_l_* = 0 would be equivalent to assuming no intermediate confounding, which is why we do not consider them.

Thus, given this particular model, we have 2 options in the presence of intermediate confounders: Either we identify the PNDE and TNIE under the assumption that β*_xm_* = 0 or we identify them under the assumption that β*_ll_* = β*_xl_* = 0. Hence, examining the significance of these parameters in an associational model for *Y* that contains all of these terms should aid in the selection of identification assumptions.

### Equivalence in assumptions

**Figure 3.**
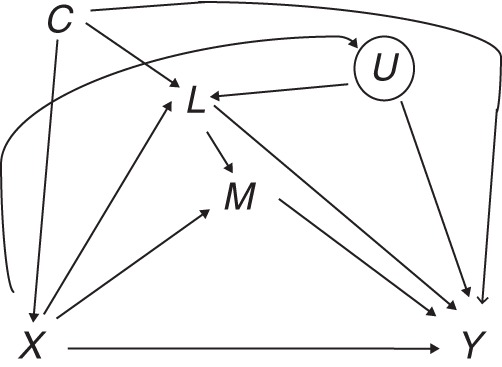
Causal diagram for exposure *X*, mediator *M*, outcome *Y*, intermediate confounder *L*, and unmeasured intermediate *L-Y* confounder *U*. The circle around *U* indicates that it is unmeasured.

As we stated above, there is an interesting difference with regard to the identifying assumptions invoked by the 2 approaches when the model involves intermediate confounders. Under the SEM, all of the error terms are assumed to be uncorrelated with each other, a scenario which would not be satisfied were the *L*-*Y* relationship affected by unmeasured confounding, given *C* and *X* (represented by *U* in Figure 3). This is not a restriction invoked by the causal inference framework (as it concerns only confounding of *X*-*Y*, *X*-*M*, and *M*-*Y*).

However, when the focus is identification of mediation effects within the SEM framework, the assumption of no *L-Y* confounding is actually not required once the parametric assumptions discussed above are made (for a justification based on the theory described by Wermuth and Cox (61), see part C of the Web Appendix and—for a simpler setting—Moerkerke et al. (62); also see Pearl (63)). Thus, there is no contradiction in fitting a SEM without assuming no *L*-*Y* confounding.

### Sensitivity analyses

It is possible to perform simple sensitivity analyses of the assumption of no unmeasured *M*-*Y* confounding by fitting SEMs that allow for $\epsilon_{y}$ and $\epsilon_{m}$ to be correlated (10, 49, 64). We extend the sensitivity analysis of Imai et al. (49) to a setting with intermediate confounders—for example,

(6) $$\left\{\begin{array}{ll} L & =\gamma_{0}+\gamma_{x}X+\epsilon_{l}\\ M & =\alpha_{0}+\alpha_{x}X+\alpha_{l}L+\epsilon_{m}\\ Y & =\beta_{0}+\beta_{x}X+\beta_{m}M+\beta_{l}L+\epsilon_{y}, \end{array}\right.$$

where, for simplicity, there are no confounders or interaction terms and the residuals are uncorrelated with the explanatory variables in their equations and have constant variance ($\text{Var(}\epsilon_{l})=\mathrm{Var}(\epsilon_{l}|X)=\sigma_{l}^{2}$, $\mathrm{Var}(\epsilon_{m})=\mathrm{Var}(\epsilon_{m}|X,L)=\sigma_{m}^{2}$, and $\mathrm{Var}(\epsilon_{y})=\mathrm{Var}(\epsilon_{y}|X,L,M)=\sigma_{y}^{2}$)) but $\epsilon_{m}$ and $\epsilon_{y}$ are correlated with $\mathrm{Corr}(\epsilon_{m},\epsilon_{y})=\mathrm{Corr}(\epsilon_{m},\epsilon_{y}|X,L,M)=\rho$. This would occur in the presence of uncontrolled *M*-*Y* confounding.

Now consider the alternative specification:

(7) $$\left\{\begin{array}{ll} L & =\gamma_{0}+\gamma_{x}X+\epsilon_{l}\\ M & =\alpha_{0}+\alpha_{x}X+\alpha_{l}L+\epsilon_{m}\\ Y & ={\beta^{\prime }}_{0}+{\beta^{\prime }}_{x}X+{\beta^{\prime }}_{l}L+{\epsilon^{\prime }}_{y}, \end{array}\right.$$

where the model for *Y* does not include *M* and $\mathrm{Var}(\epsilon_{y}^{\prime })=\mathrm{Var}(\epsilon_{y}^{\prime }|X,L)=\sigma_{y}^{\;\prime \;2}$, and $\mathrm{Corr}(\epsilon_{m},\epsilon_{y}^{\prime })=\mathrm{Corr}(\epsilon_{m},\epsilon_{y}^{\prime }|X,L)=\rho^{\prime }$. The parameters of model 6 (equation 6) are not identified because β*_m_* and ρ are collinear, whereas the parameters of model 7 (equation 7) are.

Similarly to Imai et al. (49), we focus on ρ′ and interpret it as a measure of the strength of any unmeasured *M*-*Y* confounding that would imply an indirect effect of zero. Estimating ρ′ is straightforward: Model 7 is fitted and the residuals are calculated, with their sample correlation being ${\hat{\rho }}^{\prime }$. A confidence interval for ${\hat{\rho }}^{\prime }$ is then obtained by bootstrapping (Stata code (StataCorp LP, College Station, Texas) given in Appendix 3).

## RESULTS

To illustrate the advantages of fitting SEMs when studying mediation, we analyze data on eating-disorder behaviors in adolescent girls. An adolescent eating-disorder study was carried out as part of the Avon Longitudinal Study of Parents and Children (ALSPAC), a birth cohort study of babies born between 1990 and 1992 in the South West of the United Kingdom (65). It involved data on eating-disorder behaviors collected by parental questionnaire on nearly 3,000 girls when they were around age 13.5 years. This information was used to identify 3 (standardized) latent scores for disordered eating patterns via factor analysis (66). For illustration, we use one of these latent dimensions, “bingeing or overeating,” as the outcome of interest and study whether the influence of high maternal prepregnancy body mass index (BMI; weight (kg)/height (m)^2^; coded >25 for high and ≤25 for low) is mediated by the daughter's BMI in childhood (prospectively calculated from measurements taken at about age 7 years). It is of interest to separate the effects that maternal BMI may have through and not through potentially modifiable childhood factors.

**Figure 4.**
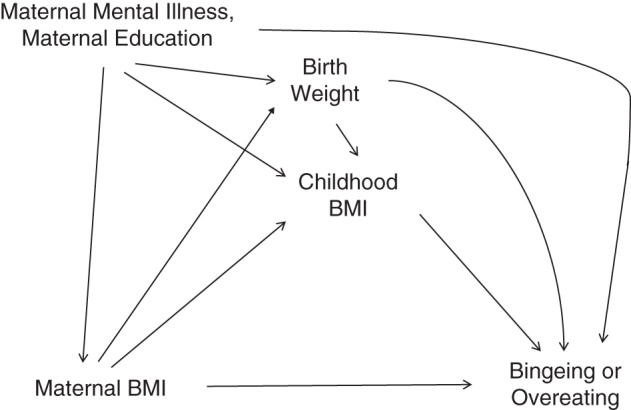
Causal diagram for the relationships between high maternal prepregnancy body mass index (BMI; weight (kg)/height (m)^2^) (*X*), birth weight (*L*), offspring childhood BMI (prospectively calculated from measurements taken at about age 7 years) (*M*), and offspring “bingeing or overeating” score, measured at around age 13.5 years (*Y*), Avon Longitudinal Study of Parents and Children, United Kingdom, 1990–2005.

The assumed causal diagram is shown in Figure 4, with maternal prepregnancy mental illness and education as background confounders (*C*_1_ and *C*_2_) and birth weight as an intermediate confounder (*L*). The appropriate extension (i.e., incorporating the mediation formula) of the G-computation formula by Monte Carlo simulation was performed via the gformula command in Stata 13 (50) (details given in Appendix 2, part A); estimation by combination was performed after fitting models by maximum likelihood in Mplus 7.11 (67) and combining the relevant estimated parameters as appropriate (details given in Appendix 2, part B). Standard errors were obtained via the bootstrap and delta methods, respectively.

Analyses are restricted to the 2,749 girls with complete data on all variables. Table 1 characterizes the data and shows marginal and partial correlations. “Bingeing or overeating” is both marginally and conditionally correlated with all other variables except maternal education, while maternal BMI (but not childhood BMI) is correlated with birth weight.

**Table 1.** Mean Values/Percentages and Marginal (Above Main Diagonal) and Partial (Below Main Diagonal) Correlations for Variables Used in an Analysis of Eating-Disorder Behaviors Among Adolescent Girls (*n* = 2,749), Avon Longitudinal Study of Parents and Children, United Kingdom, 1990–2005^a^

Variable	Symbol	Mean (SD)	%	Correlation					
				Bingeing or Overeating	Childhood BMI^b^	Birth Weight	High Maternal BMI	Low Maternal Education	Poor Maternal Mental Health
Bingeing or overeating	*Y*	0.00 (1.00)		1	0.33^c^	0.05^c^	0.06^c^	−0.01	0.11^c^
Childhood BMI^d^	*M*	−0.02 (0.99)		0.34^c^	1	−0.02	0.26^c^	0.10^c^	−0.02
Birth weight^e^	*L*	0.10 (0.92)		0.05^c^	0.01	1	0.12^c^	−0.04	−0.04
High maternal BMI^f,g^	*X*		19	0.17^c^	0.31^c^	0.13^c^	1	0.17^c^	−0.03
Low maternal education^g,h^	*C*_1_		55	0.04	0.13^c^	−0.03^c^	0.20^c^	1	0.04
Poor maternal mental health^g^	*C*_2_		13	0.11^c^	0.01	−0.03	−0.01	0.04	1

Abbreviations: BMI, body mass index; SD, standard deviation.

a Information on maternal education, prepregnancy BMI, and history of mental illness was obtained from postal questionnaires administered during pregnancy. Birth weight was measured at the time of birth. Childhood BMI was prospectively calculated from measurements taken at about age 7 years.

b Weight (kg)/height (m)^2^.

c *P* < 0.05.

d Childhood BMI was age-standardized (leading to a standardized score). Because of missing values on other variables, its mean and SD were not exactly 0 and 1.

e Birth weight was internally standardized using the complete sample (leading to a standardized score). Because of missing values on other variables, its mean and SD were not exactly 0 and 1.

f Maternal prepregnancy BMI was dichotomized (<25, low; ≥25, high).

g Polychoric (or tetrachoric) correlations are reported when calculations involved this variable.

h Maternal education was dichotomized: “no high school” versus “at least high school.”

Table 2 shows the estimated coefficients for the conditional expectation of *Y* expressed without any of the parametric constraints needed for identification in the presence of intermediate confounders. In particular, we allowed interactions between *X* and *M*, *L* and *M*, and nonlinearities in *L* and *M*. It appears that there is little evidence to reject β*_xm_* = 0 (*P* = 0.76), while the evidence for β*_xl_* and β*_ll_* being nonzero is greater (*P* = 0.08 and *P* = 0.01, respectively), suggesting that the Robins and Greenland assumption may be more plausible in this example. We nevertheless report the estimates of the mediation effects obtained under both assumptions in Table 3 (see also Web Table 1). The results suggest a strong mediated effect of high maternal BMI on “bingeing or overeating” via childhood BMI, with a smaller direct effect capturing all other pathways. It appears therefore that more than 60% of the total effect of maternal overweight is transmitted via the daughter's own size in childhood and not via other pathways, including birth weight, implicating a contribution of childhood environmental factors. Table 3 also highlights the closeness of the results obtained using Monte Carlo G-computation and G-computation via estimation by combination; however, this required the size of the Monte Carlo sample to be increased to 100,000.

**Table 2.** Estimated Coefficients From a Regression Model for “Bingeing or Overeating” Among Adolescent Girls (*n* = 2,749), Avon Longitudinal Study of Parents and Children, United Kingdom, 1990–2005

Variable	Symbol	Parameter	Estimate (SE)	*P* Value
High maternal BMI^a^	*X*	β*_x_*	0.068 (0.050)	0.18
Childhood BMI score	*M*	β*_m_*	0.312 (0.021)	<0.001
Childhood BMI score squared	*M*^2^	β*_mm_*	0.043 (0.012)	<0.001
Birth weight score	*L*	β*_l_*	0.034 (0.022)	0.13
Birth weight score squared	*L*^2^	β*_ll_*	0.032 (0.012)	0.01
High maternal BMI × birth weight	*XL*	β*_xl_*	0.078 (0.045)	0.08
High maternal BMI × child BMI	*XM*	β*_xm_*	0.014 (0.045)	0.76
Low maternal education	*C*_1_	β*_c_*_1_	−0.011 (0.036)	0.76
Poor maternal mental health	*C*_2_	β*_c_*_2_	0.207 (0.054)	<0.001

Abbreviations: BMI, body mass index; SE, standard error.

a Weight (kg)/height (m)^2^.

**Table 3.** Estimation of the Total Effect of High Maternal BMI on “Bingeing or Overeating” Among Adolescent Girls (*n* = 2,749) and of the Effects Mediated and Not Mediated by Childhood BMI (Estimation by Monte Carlo Simulation vs. Estimation by Combination), Avon Longitudinal Study of Parents and Children, United Kingdom, 1990–2005

Model and Estimand	Estimation Method and Estimate (SE)	
	Monte Carlo G-Computation^a^	Estimation by Combination^b^
Model 1^c^		
TCE	0.287 (0.052)	0.287 (0.049)
PNDE	0.102 (0.050)	0.103 (0.047)
TNIE	0.185 (0.021)	0.184 (0.019)
CDE(0)	0.104 (0.050)	0.103 (0.047)
Model 2^d^		
TCE	0.297 (0.052)	0.297 (0.049)
PNDE	0.102 (0.051)	0.103 (0.051)
TNIE	0.195 (0.031)	0.194 (0.028)
CDE(0)	0.105 (0.049)	0.105 (0.049)

Abbreviations: CDE, controlled direct effect; PNDE, pure natural direct effect; SE, standard error; TCE, total causal effect; TNIE, total natural indirect effect.

a Estimation by G-computation via Monte Carlo simulation was carried out using the gformula command (50) in Stata 13, with an enlarged Monte Carlo sample of 100,000 to increase agreement with closed-form results (see Appendix 2, part A); SEs were estimated via bootstrap.

b Estimation by combination was carried out by combining the maximum likelihood estimates of the relevant structural equation model parameters obtained in Mplus, version 7.11 (see Appendix 2, part B); SEs were estimated via the delta method.

c Model 1 follows the Robins and Greenland assumption (11) that there is no interaction between *X* and *M* at the individual level in their effects on *Y*. The model was specified as follows. The equation for “bingeing or overeating” (*Y*) included childhood BMI (*M*; linear and quadratic terms), high maternal BMI (*X*; binary), birth weight (*L*; linear and quadratic terms), the interaction between high maternal BMI and birth weight, maternal education (*C*_1_; binary), and prepregnancy mental health (*C*_2_; binary). The equation for childhood BMI included high maternal BMI (binary), birth weight (linear term), the interaction between high maternal BMI and birth weight, maternal education (binary), and prepregnancy mental health (binary). The equation for birth weight included high maternal BMI (binary), maternal education (binary), and prepregnancy mental health (binary).

d Model 2 follows the Petersen et al. assumption (27) that (conditional on *C*) the CDE does not vary with *M*(0). The model was specified as follows. The equation for “bingeing or overeating” included childhood BMI (linear and quadratic terms), high maternal BMI (binary), birth weight (linear term), the interaction between high maternal BMI and childhood BMI, maternal education (binary), and prepregnancy mental health (binary). The equation for childhood BMI included high maternal BMI (binary), birth weight (linear term), the interaction between high maternal BMI and birth weight, maternal education (binary), and prepregnancy mental health (binary). The equation for birth weight included high maternal BMI (binary), maternal education (binary), and prepregnancy mental health (binary).

Sensitivity analyses show that a noncausal residual correlation between childhood BMI and “bingeing or overeating” would have to be very large, at least equal to 0.324 (95% confidence interval: 0.287, 0.361), to remove the path mediated by childhood BMI.

## DISCUSSION

We have reviewed 2 alternative approaches to the study of mediation in settings with intermediate confounding. The one emerging from the SEM framework has a long tradition in the social sciences and uses definitions of direct and indirect effects that are intuitive but are embedded within simple linear models. In contrast, the approach proposed within the causal inference literature is general, as it compares expected potential outcomes without reference to any particular model.

We have extended work done by others (10, 13, 45, 49, 64) in deriving closed-form solutions to the identification equations for the causal inference estimands for general linear SEMs that include intermediate confounders. This has helped in clarifying the parametric assumptions needed for identification—and the consequent advantages of examining certain regression parameters, justifying the relaxation of the assumption of no *L*-*Y* unmeasured confounders made by the causal inference school and extending sensitivity analyses of unmeasured *M*-*Y* confounding. These results are novel and should help analysts investigating mediation in the presence of intermediate confounding. Although these results are restricted to settings that can be modeled with systems of linear equations, the insights gained here should also apply more generally, given the approximate closed-form expressions recently derived for binary outcomes and mediators (31, 68) and the recent nonparametric identifying constraints involving *L*-*M* interactions (29, 64).

## Supplementary Material

Web Material

## APPENDIX 1

Estimation by Combination for a More General Linear SEM

Consider the following linear structural equation model (SEM):

(8) $$\left\{ \begin{array}{c} \;L=\gamma_{0}+\gamma_{x}X+\gamma_{c}C+\epsilon_{l}\\ M=\alpha_{0}+\alpha_{x}X+\alpha_{l}L+\alpha_{c}C+\alpha_{xl}XL+\epsilon_{m}\\ \;Y=\beta_{0}+\beta_{x}X+\beta_{l}L+\beta_{ll}L^{2}+\beta_{m}M+\beta_{mm}M^{2}+\beta_{c}C+\beta_{xl}XL+\beta_{xm}XM+\epsilon_{y}, \end{array}\right.$$

where the residual terms are uncorrelated with each other and the endogenous variables and have variances $\sigma_{l}^{2}$, $\sigma_{m}^{2}$, and $\sigma_{y}^{2}$, respectively. (Note that these variances are assumed to be constant, so that $\mathrm{Var}(\epsilon_{l}|X,C)=\sigma_{l}^{2}$, $\mathrm{Var}(\epsilon_{m}|X,L,C)=\sigma_{m}^{2}$, and $\mathrm{Var}(\epsilon_{y}|X,L,M,C)=\sigma_{y}^{2}$.)

For this model, the expression for the pure natural direct effect (PNDE) (see Web Appendix, part B),

(9) $$\begin{array}{rl} & \int_{c}\{\int_{l^{\;\prime }}\int_{m}\int_{l}\left\{E(Y|X=1,M=m,L=l,C=c)\;f_{L}(l|X=1,C=c\right)\\ & \;\;-E(Y|X=0,M=m,L=l,C=c)\;f_{L}(l|X=0,C=c)\}\;dl\\ & \;\;\times \;f_{M}(m|L=l^{\;\prime },X=0,C=c)\;f_{L}(l^{\;\prime }|X=0,C=c)\;dm\;dl^{\;\prime }\}\;f_{C}(c)\;dc, \end{array}$$

can be written in closed form. Consider first its inner component:

$$\begin{array}{rl} & \int_{l}\left\{E(Y|X=1,M=m,L=l,C=c)\;f_{L}(l|X=1,C=c\right)\\ & \;-E(Y|X=0,M=m,L=l,C=c)\;f_{L}(l|X=0,C=c)\}\;dl. \end{array}$$

This is equal to

(10) $$\begin{array}{rl} & \left\{\beta_{0}+\beta_{x}+(\beta_{l}+\beta_{xl})\bar{L_{1}}(c)+\beta_{ll}\bar{L_{1}^{2}}(c)+\beta_{m}m+\beta_{mm}m^{2}+\beta_{c}c+\beta_{xm}m\right\}\\ & \;-\{\beta_{0}+\beta_{l}\bar{L_{0}}(c)+\beta_{ll}\bar{L_{0}^{2}}(c)+\beta_{m}m+\beta_{mm}m^{2}+\beta_{c}c\}\\ & \;\;=\beta_{x}+\beta_{l}(\bar{L_{1}}(c)-\bar{L_{0}}(c))+\beta_{ll}(\bar{L_{1}^{2}}(c)-\bar{L_{0}^{2}}(c))+\beta_{xl}\bar{L_{1}}(c)+\beta_{xm}m, \end{array}$$

where

$$\begin{array}{rl} & \bar{L_{x}}(c)=E(L|X=x,C=c)=\gamma_{0}+\gamma_{x}x+\gamma_{c}c\\ & \bar{L_{x}^{2}}(c)=E(L^{2}|X=x,C=c)=(\bar{L_{x}}(c){)}^{2}+\sigma_{l}^{2}\\ & \bar{L_{1}}(c)-\bar{L_{0}}(c)=\gamma_{x}\\ & \bar{L_{1}^{2}}(c)-\bar{L_{0}^{2}}(c)=(\bar{L_{1}}(c){)}^{2}-(\bar{L_{0}}(c){)}^{2}=\gamma_{x}^{2}+2\gamma_{x}(\gamma_{0}+\gamma_{c}c). \end{array}$$

Let

$$A(c)=\beta_{x}+\beta_{l}\gamma_{x}+\beta_{ll}\{\gamma_{x}^{2}+2\gamma_{x}(\gamma_{0}+\gamma_{c}c)\}+\beta_{xl}(\gamma_{0}+\gamma_{x}+\gamma_{c}c).$$

Writing equation 10 as *A*(*c*) + β*_xm_m*, we can rewrite equation 9 as

$$\begin{array}{rl} & \int_{c}\int_{l^{\;\prime }}\int_{m}(A(c)+\beta_{xm}m)\;f_{M}(m|L=l^{\;\prime },X=0,C=c)\;f_{l}(l^{\;\prime }|X=0,C=c)\;f_{C}(c)\;dm\;dl^{\;\prime }\;dc\\ & \;=\int_{c}\{A(c)+\beta_{xm}\bar{M_{0}}(c)\}\;f_{C}(c)\;dc\\ & \;=\bar{A}+\beta_{xm}\bar{\bar{M_{0}}}, \end{array}$$

where

$$\begin{array}{rl} \bar{M_{x}}(c) & =E(M|X=x,C=c)=\alpha_{0}+\alpha_{x}x+(\alpha_{l}+\alpha_{xl}x)\bar{L_{x}}(c)+\alpha_{c}c\\ \bar{M_{0}}(c) & =\alpha_{0}+\alpha_{l}(\gamma_{0}+\gamma_{c}c)+\alpha_{c}c\\ \bar{A} & =\int A(c)\;f_{c}(c)\;dc\\ & =\beta_{x}+\beta_{l}\gamma_{x}+\beta_{ll}\{\gamma_{x}^{2}+2\gamma_{x}(\gamma_{0}+\gamma_{c}\mu_{c})\}+\beta_{xl}(\gamma_{0}+\gamma_{x}+\gamma_{c}\mu_{c})\\ \bar{\bar{M_{0}}} & =\int \bar{M_{0}}(c)\;f_{c}(c)\;dc\\ & =\alpha_{0}+\alpha_{l}(\gamma_{0}+\gamma_{c}\mu_{c})+\alpha_{c}\mu_{c}. \end{array}$$

Thus, equation 9 becomes

$$\beta_{x}+\beta_{l}\gamma_{x}+\beta_{ll}\{\gamma_{x}^{2}+2\gamma_{x}(\gamma_{0}+\gamma_{c}\mu_{c})\}+\beta_{xl}(\gamma_{0}+\gamma_{x}+\gamma_{c}\mu_{c})+\beta_{xm}\{\alpha_{0}+\alpha_{l}(\gamma_{0}+\gamma_{c}\mu_{c})+\alpha_{c}\mu_{c}\}.$$

If the model is correctly specified and if, additionally, the assumptions of no interference, strong consistency, and conditional exchangeability are met and one of the parametric assumptions described in the text is met, then this expression can be interpreted as the PNDE. However, note that the additional parametric assumptions constrain some of the parameters above to be zero, and thus the expression simplifies.

Similar calculations for CDE(*m*), the controlled direct effect (CDE) of *X* on *Y* when *M* is controlled at *m* (see Web Appendix, part B), lead to

$$\begin{array}{rl} \mathrm{CDE}(m) & =\bar{A}+\beta_{xm}m\\ & =\beta_{x}+\beta_{l}\gamma_{x}+\beta_{ll}\{\gamma_{x}^{2}+2\gamma_{x}(\gamma_{0}+\gamma_{c}\mu_{c})\}+\beta_{xl}(\gamma_{0}+\gamma_{x}+\gamma_{c}\mu_{c})+\beta_{xm}m, \end{array}$$

with the interpretation as CDE(*m*) being justified if the model is correctly specified and if the appropriate assumptions (no interference, consistency, conditional exchangeability) are met; note that the parametric restrictions described in the text are not required for this estimand.

Finally, for the total natural indirect effect (TNIE) (see Web Appendix, part B), we have the expression

$$\begin{array}{rl} & \int_{c}\int_{l^{\;\prime }}\int_{m}\int_{l}E(Y|X=1,M=m,L=l,C=c)\;f_{L}(l|X=1,C=c)\\ & \;\times \{\;f_{M}(m|X=1,L=l^{\;\prime },C=c)\;f_{L}(l^{\;\prime }|X=1,C=c)\\ & \;-\;f_{M}(m|X=0,L=l^{\;\prime },C=c)\;f_{L}(l^{\;\prime }|X=0,C=c)\}\;f_{C}(c)\;dl\;dm\;dl^{\;\prime }\;dc, \end{array}$$

which can be rewritten as

(11) $$\int_{c}\{(\beta_{m}+\beta_{xm})(\bar{M_{1}}(c)-\bar{M_{0}}(c))+\beta_{mm}(\bar{M_{1}^{2}}(c)-\bar{M_{0}^{2}}(c))\}\;f_{C}(c)\;dc,$$

where

$$\begin{array}{rl} \bar{M_{1}}(c)-\bar{M_{0}}(c) & =\alpha_{x}+\alpha_{xl}(\gamma_{0}+\gamma_{x}+\gamma_{c}c)+\alpha_{l}\gamma_{x}\\ \bar{M_{1}^{2}}(c)-\bar{M_{0}^{2}}(c) & =E(M^{2}|X=1,C=c)-E(M^{2}|X=0,C=c)\\ & =\{\bar{M_{1}}(c){\}}^{2}-\{\bar{M_{0}}(c){\}}^{2}+\mathrm{Var}(M|X=1,C=c)-\mathrm{Var}(M|X=0,C=c)\\ & =\{\alpha_{0}+\alpha_{x}+(\alpha_{l}+\alpha_{xl})(\gamma_{0}+\gamma_{x}+\gamma_{c}c)+\alpha_{c}c{\}}^{2}-\{\alpha_{0}+\alpha_{l}(\gamma_{0}+\gamma_{c}c)+\alpha_{c}c{\}}^{2}\\ & \;+(\alpha_{l}+\alpha_{xl}{)}^{2}\sigma_{l}^{2}+\sigma_{m}^{2}-(\alpha_{l}^{2}\sigma_{l}^{2}+\sigma_{m}^{2})\\ & =\{\alpha_{0}+\alpha_{l}(\gamma_{0}+\gamma_{x}+\gamma_{c}c)+\alpha_{c}c{\}}^{2}\\ & \;+2\{\alpha_{0}+\alpha_{l}(\gamma_{0}+\gamma_{x}+\gamma_{c}c)+\alpha_{c}c\}\{\alpha_{x}+\alpha_{xl}(\gamma_{0}+\gamma_{x}+\gamma_{c}c)\}\\ & \;+(2\alpha_{l}+\alpha_{xl})\alpha_{xl}\sigma_{l}^{2}. \end{array}$$

Thus, equation 11 can be rewritten as

$$\begin{array}{rl} & (\beta_{m}+\beta_{xm})\{\alpha_{x}+\alpha_{xl}(\gamma_{0}+\gamma_{x}+\gamma_{c}\mu_{c})+\alpha_{l}\gamma_{x}\}+\beta_{mm}(\{\alpha_{x}+\alpha_{l}\gamma_{x}+\alpha_{xl}(\gamma_{0}+\gamma_{x}){\}}^{2}\\ & \;+2(\alpha_{0}+\alpha_{l}\gamma_{0})\{\alpha_{x}+\alpha_{l}\gamma_{x}+\alpha_{xl}(\gamma_{0}+\gamma_{x})\}\\ & \;+2[(\alpha_{0}+\alpha_{l}\gamma_{0})\alpha_{xl}\gamma_{c}+\{\alpha_{x}+\alpha_{l}\gamma_{x}+\alpha_{xl}(\gamma_{0}+\gamma_{x})\}(\alpha_{c}+\alpha_{l}\gamma_{c}+\alpha_{xl}\gamma_{c})]\mu_{c}\\ & \;+\{2(\alpha_{c}+\alpha_{l}\gamma_{c})+\alpha_{xl}\gamma_{c}\}\alpha_{xl}\gamma_{c}(\mu_{c}^{2}+\sigma_{c}^{2})+(2\alpha_{l}+\alpha_{xl})\alpha_{xl}\sigma_{l}^{2}), \end{array}$$

where $\sigma_{c}^{2}$ is the variance of *C*.

Again, this can be interpreted as the TNIE if the model is correctly specified and if, additionally, the assumptions of no interference, strong consistency, and conditional exchangeability are met and one of the parametric assumptions is met. Note again that the additional parametric assumptions constrain some of the parameters above to be zero, simplifying the expression.

## APPENDIX 2

G-Computation in Stata and Mplus

*y*: dependent variable
*x*: exposure
*m*: mediator
*l*: intermediate confounder
*c*_1: first baseline confounder
*c*_2: second baseline confounder
*m*2: *m*^2^
*l*2: *l*^2^
*xl*: *x* × *l*
*xm*: *x* × *m*

### A. G-computation by Monte Carlo simulations using Stata

To implement G-computation by Monte Carlo simulation, we have used the user-written command gformula. The syntax used was as follows (for more details, refer to Daniel et al. (50)):

1. Model 1 (Robins and Greenland's identifying assumptions (11)):

  #delimit ;

  gformula y x m m2 l l2 c1 c2 xl,

  mediation outcome(y) exposure(x) mediator(m)

  post_confs(l) base_confs(c1 c2)

  obe control( m:0)

  commands(y:regress, m:regress, l:regress)

  equations(y:x m m2 l l2 c1 c2 xl, m:x l c1 c2 xl, l:x c1 c2)

  derived(m2 l2 xl) derrules(m2:m*m,l2:l*l, xl:x*l)

  minsim samples(1000) moreMC simulations(100000) replace seed(79);

  #delimit cr

2. Model 2 (Petersen et al.'s identifying assumptions (27)):

  #delimit ;

  gformula y x m m2 l l2 c1 c2 xl xm,

  mediation outcome(y) exposure(x) mediator(m)

  post_confs(l) base_confs(c1 c2)

  obe control( m:0)

  commands(y:regress, m:regress, l:regress)

  equations(y:x m m2 l c1 c2 xm, m:x l c1 c2 xl, l:x c1 c2)

  derived(m2 l2 xl xm) derrules(m2:m*m,l2:l*l, xl:x*l, xm:x*m)

  minsim samples(1000) moreMC simulations(100000) replace seed(79);

  #delimit cr

### B. G-computation via estimation by combination using Mplus

The implementation with 2 confounders requires an extension of the expressions given in Appendix 1.

Let μ*_c_*_1_ and μ*_c_*_2_ be the mean values of the 2 confounders, $\sigma_{c1}^{2}$ and $\sigma_{c1}^{2}$ their variances, and $\sigma_{12}$ their covariance. Also let

$$\begin{array}{rl} L_{0} & =\gamma_{0}+(\gamma_{c1}\mu_{c1}+\gamma_{c2}\mu_{c2})\\ L_{1} & =L_{0}+\gamma_{x}\\ P_{1} & =\alpha_{0}+\alpha_{l}\gamma_{0}\\ P_{2} & =\alpha_{x}+\alpha_{l}\gamma_{x}+\alpha_{xl}(\gamma_{0}+\gamma_{x})\\ \bar{A} & =\beta_{x}+\beta_{l}\gamma_{x}+\beta_{ll}\{\gamma_{x}^{2}+2\gamma_{x}L_{0}\}+\beta_{xl}L_{1}\\ \bar{\bar{M_{0}}} & =\{\alpha_{0}+\alpha_{l}L_{0}+\alpha_{c1}\mu_{c1}+\alpha_{c2}\mu_{c2}\}\\ P_{c1} & =(\alpha_{c1}+\alpha_{l}\gamma_{c1}+\alpha_{xl}\gamma_{c1})\mu_{c1}\\ P_{c2} & =(\alpha_{c2}+\alpha_{l}\gamma_{c2}+\alpha_{xl}\gamma_{c2})\mu_{c2}. \end{array}$$

Then,

$$\begin{array}{rl} \mathrm{CDE}(m) & =\bar{A}+\beta_{xm}m\\ \mathrm{PNDE} & =\bar{A}+\beta_{xm}\bar{\bar{M_{0}}}\\ \mathrm{TNIE} & =(\beta_{m}+\beta_{xm})\{\alpha_{x}+\alpha_{xl}L_{1}+\alpha_{l}\gamma_{x}\}\\ & \;+\beta_{mm}(P_{2}^{2}+2P_{1}P_{2}+2[P_{1}\alpha_{xl}\gamma_{c1}\mu_{c1}+P_{2}P_{c1}]\mu_{c1}+2[P_{1}\alpha_{xl}\gamma_{c2}\mu_{c2}+P_{2}P_{c2}]\mu_{c2}\\ & \;+[2(\alpha_{c1}+\alpha_{l}\gamma_{c1})+\alpha_{xl}\gamma_{c1}]\alpha_{xl}\gamma_{c1}(\mu_{c1}^{2}+\sigma_{c1}^{2})+[2(\alpha_{c2}+\alpha_{l}\gamma_{c2})+\alpha_{xl}\gamma_{c2}]\alpha_{xl}\gamma_{c2}(\mu_{c2}^{2}+\sigma_{c2}^{2})\\ & \;+([2(\alpha_{c1}+\alpha_{l}\gamma_{c1})+\alpha_{xl}\gamma_{c2}]\alpha_{xl}\gamma_{c1}+[2(\alpha_{c2}+\alpha_{l}\gamma_{c2})+\alpha_{xl}\gamma_{c1}]\alpha_{xl}\gamma_{c2})(\mu_{c1}\mu_{c2}+\sigma_{12})\\ & \;+(2\alpha_{l}+\alpha_{xl})\alpha_{xl}\sigma_{l}^{2}). \end{array}$$

The code below is for Mplus, version 7.11 (67), where we use the labeling options to identify the relevant parameters.

1. Model 1 (Robins and Greenland's identifying assumptions (11)):

   TITLE: Model 1

 DATA: FILE IS “……”;

 Format is free;

 LISTWISE=ON;

  VARIABLE: NAMES ARE id y x m m2 l l2 c_1 c_2 xl xm;

 USEV ARE y x m m2 l l2 c_1 c_2 xl;

 MISSING ARE .;

 IDVARIABLE= id;

  MODEL:

 [y] (beta0);

 y ON x (betax);

 y ON m (betam);

 y ON m2 (betamm);

 y ON l (betal);

 y ON l2 (betall);

 y ON c_1 (betac1);

 y ON c_2 (betac2);

 y ON xl (betaxl);

 [m] (alpha0);

 m ON x (alphax);

 m ON l (alphal);

 m ON c_1 (alphac1);

 m ON c_2 (alphac2);

 m ON xl (alphaxl);

 m (sigma2m);

 [l] (gamma0);

 l ON x (gammax);

 l ON c_1 (gammac1);

 l ON c_2 (gammac2);

 l (sigma2l);

 [c_1] (muc1);

 [c_2] (muc2);

 c_1 (sigma2c1);

 c_2 (sigma2c2);

 c_1 WITH c_2 (covc1c2);

 MODEL CONSTRAINT:

 !this command lists all the terms used for the calculations

 !and gives them starting values:

 NEW (betaxm*0 L0*.1 L1*.1 P1*.1 P2*.1 P_c1*.1 P_c2*.1

 A_bar*.1 M_barbar_0*.1 cde0*.1 pnde*.1 tnie*.1 tce*0.1 );

  !this is to remind ourselves of the Robins and Greenland assumption

  ! while using the general expressions

    betaxm=0;

  !for CDE(0)

  L0 = gamma0+(gammac1*muc1+gammac2*muc2);

  L1 = gamma0+gammax+(gammac1*muc1+gammac2*muc2);

  A_bar=betax+betal*gammax+betall*(gammax*gammax+2*gammax*L0)+betaxl*L1;

  cde0=A_bar+betaxm*0;

  !for PNDE

  M_barbar_0=alpha0+alphal*L0+(alphac1*muc1+alphac2*muc2);

  pnde=A_bar+betaxm*M_barbar_0;

  !for TNIE

  P1=alpha0+alphal*gamma0;

  P2= (alphax +alphal*gammax+alphaxl*(gamma0+gammax));

  P_c1=(alphac1+alphal*gammac1+alphaxl*gammac1)*muc1;

  P_c2=(alphac2+alphal*gammac2+alphaxl*gammac2)*muc2;

  tnie=(betam+betaxm)*(alphax+alphaxl*L1+gammax*alphal)

  + betamm*(P2*P2+2*P1*P2

  +2*(P1*alphaxl*gammac1*muc1+P2*P_c1)

  +2*(P1*alphaxl*gammac2*muc2+P2*P_c2)

  +(2*(alphac1+alphal*gammac1)+alphaxl*gammac1)*alphaxl*gammac1*

  (muc1*muc1+sigma2c1)

  +(2*(alphac2+alphal*gammac2)+alphaxl*gammac2)*alphaxl*gammac2*

  (muc2*muc2+sigma2c2)

  +( (2*(alphac1+alphal*gammac1)+alphaxl*gammac1)*alphaxl*gammac2

  +(2*(alphac2+alphal*gammac2)+alphaxl*gammac2)*alphaxl*gammac1

  )*(muc1*muc2+covc1c2)

  +(2*alphal+alphaxl)*alphaxl*sigma2l

  );

  tce=tnie+pnde;

  OUTPUT: SAMPSTAT ;

2. Model 2 (Petersen et al.'s identifying assumptions (27)):

   TITLE: Model 2

 DATA: FILE IS “ ……..dat”;

 Format is free;

 LISTWISE=ON;

 VARIABLE: NAMES ARE id y x m m2 l l2 c_1 c_2 xl xm;

  USEV ARE y x m m2 l c_1 c_2 xm xl;

 MISSING ARE .;

 IDVARIABLE= id;

  MODEL:

 [y] (beta0);

 y ON x (betax);

 y ON m (betam);

 y ON m2 (betamm);

 y ON l (betal);

 y ON c_1 (betac1);

 y ON c_2 (betac2);

 y ON xm (betaxm);

 [m] (alpha0);

 m ON x (alphax);

 m ON l (alphal);

 m ON c_1 (alphac1);

 m ON c_2 (alphac2);

 m ON xl (alphaxl);

  m (sigma2m);

  [l] (gamma0);

 l ON x (gammax);

 l ON c_1 (gammac1);

 l ON c_2 (gammac2);

 l (sigma2l);

 [c_1] (muc1);

 [c_2] (muc2);

 c_1 (sigma2c1);

 c_2 (sigma2c2);

 c_1 WITH c_2 (covc1c2);

 MODEL CONSTRAINT:

 NEW (betall*0 betaxl*0 L0*.1 L1*.1

 P1*.1 P2*.1 P_c1*.1 P_c2*.1

 A_bar*.1 M_barbar_0*.1

 cde0*.1 pnde*.1 tnie*.1 tce*0.1 );

 !this is to remind us of the Petersen et al assumptions

 ! while using the general expressions

 betall=0;

 betaxl=0;

 !for CDE(0)

 L0 = gamma0+(gammac1*muc1+gammac2*muc2);

 L1 = gamma0+gammax+(gammac1*muc1+gammac2*muc2);

 A_bar=betax+betal*gammax+betall*(gammax*gammax+2*gammax*L0)+betaxl*L1;

 cde0=A_bar+betaxm*0;

 !for PNDE

 M_barbar_0=alpha0+alphal*L0+(alphac1*muc1+alphac2*muc2);

 pnde=A_bar+betaxm*M_barbar_0;

 !for TNIE

 P1=alpha0+alphal*gamma0;

 P2= (alphax +alphal*gammax+alphaxl*(gamma0+gammax));

 P_c1=(alphac1+alphal*gammac1+alphaxl*gammac1)*muc1;

 P_c2=(alphac2+alphal*gammac2+alphaxl*gammac2)*muc2;

 tnie=(betam+betaxm)*(alphax+alphaxl*L1+gammax*alphal)

 + betamm*(P2*P2+2*P1*P2

 +2*(P1*alphaxl*gammac1*muc1+P2*P_c1)

 +2*(P1*alphaxl*gammac2*muc2+P2*P_c2)

 +(2*(alphac1+alphal*gammac1)+alphaxl*gammac1)*alphaxl*gammac1*

 (muc1*muc1+sigma2c1)

 +(2*(alphac2+alphal*gammac2)+alphaxl*gammac2)*alphaxl*gammac2*

(muc2*muc2+sigma2c2)

+( (2*(alphac1+alphal*gammac1)+alphaxl*gammac1)*alphaxl*gammac2

+(2*(alphac2+alphal*gammac2)+alphaxl*gammac2)*alphaxl*gammac1

)*(muc1*muc2+covc1c2)

+(2*alphal+alphaxl)*alphaxl*sigma2l

);

tce=tnie+pnde;

OUTPUT: SAMPSTAT ;

## APPENDIX 3

Stata—Sensitivity Analysis

Sensitivity analyses were carried out using the ado file called sens_rho.ado, outlined below. It fits a posited structural equation model (SEM), with an equation each for *Y*, *M*, and *L*. In this example, it fits a model consonant with Robins and Greenland's assumption (11). Note that the model for *Y* does not include *M* or any function of *M* among its explanatory variables, in order to allow for a correlation between the error terms of the *Y* and *M* equations.

program define sens_rho, rclass

version 13

preserve

cap matrix drop Psi

sem (y <- x l xl l2 c_1 c_2) (l<- x c_1 c_2) (m <- x l xl l2 c_1 c_2), \\\

nocapslatent cov(e.y*e.m)

qui estat framework, fitted

matrix Psi=r(Psi)

matrix list Psi

scalar rho_dash=(Psi[3,1])/(sqrt(Psi[1,1]*Psi[3,3]))

scalar list rho_dash

return scalar rho=rho_dash

restore

end

It is best to check that sens_rho.ado picks the right elements of the error term's variance-covariance matrix by running the program once:

. sens_rho

Then, one needs to type

. bootstrap rho_dash=r(rho), reps(1000) saving(sens_rho,replace):sens_rho

. estat bootstrap, all

to run the Stata bootstrap command with 1,000 replications and see the results.
