# Supplementary material for: Mediation Analysis With Intermediate Confounding: Structural Equation Modeling Viewed Through the Causal Inference Lens
Source: Am J Epidemiol. 2014 Dec 11;181(1):64–80. doi: 10.1093/aje/kwu239 (PMC4383385; doi:10.1093/aje/kwu239)
Supplement: Web Material [file supp_181_1_64__index.html]

Mediation Analysis With Intermediate Confounding: Structural Equation Modeling Viewed Through the Causal Inference Lens — Mediation Analysis With Intermediate Confounding: Structural Equation Modeling Viewed Through the Causal Inference Lens — Web Material 

# Mediation Analysis With Intermediate Confounding: Structural Equation Modeling Viewed Through the Causal Inference Lens

## Web Material

Web Material

**Files in this Data Supplement:**

- Web Material - Pdf file
