# Supplementary material for: Mediation Analysis With Intermediate Confounding: Structural Equation Modeling Viewed Through the Causal Inference Lens
Source: Am J Epidemiol. 2014 Dec 11;181(1):64–80. doi: 10.1093/aje/kwu239 (PMC4383385; doi:10.1093/aje/kwu239)
Supplement: Web Material [file supp_kwu239_kwu239supp.pdf]

Mediation analysis with intermediate confounding: structural equation modeling viewed through the causal inference lens
De Stavola *et al.*

WEB APPENDIX

Part A: Identification equations for continuous Y and M in the absence of intermediate confounders

For continuous M, expression (2) in the main text becomes:

$$\sum_c \left\{ \int_m \{E(Y|X=1,M=m,C=c) - E(Y|X=0,M=m,C=c)\} \times f_M(m|X=0,C=c)\,dm \right\} Pr(C=c)$$

and expression (3) becomes:

$$\sum_c \left\{ \int_m E(Y|X=1,M=m,C=c)\, f_M(M=m|X=1,C=c)\,dm - \int_m E(Y|X=1,M=m,C=c)\, f_M(M=m|X=0,C=c)\,dm \right\} Pr(C=c).$$

Part B: Identification in the presence of intermediate confounders

If the relevant no interference, consistency and CE assumptions are met, *CDE*(m) is identified, for discrete C and L, by:

$$\sum_c \left\{ \sum_l E\{(Y|X=1,M=m,C=c,L=l)\, Pr(L=l|X=1,C=c) - E(Y|X=0,M=m,C=c,L=l)\, Pr(L=l|X=0,C=c) \right\} Pr(C=c).$$

For continuous C and L the expression above becomes:

$$\int_c \left\{ \int_l E\{(Y|X=1,M=m,C=c,L=l)\, f_L(l|X=1,C=c)dl - \int_l E(Y|X=0,M=m,C=c,L=l)\, f_L(l|X=0,C=c)dl \right\} f_C(c)dc.$$

If the relevant no interference, consistency and CE assumptions are met, together with one of the two possible parametric restrictions (see Appendix 2 in the main text), *PNDE* is identified, for discrete C, M

and L, by:

$$\sum_c \left\{ \sum_{l',m,l} \{E\left(Y|X=1, M=m, L=l, C=c\right) Pr\left(L=l|X=1, C=c\right) - E\left(Y|X=0, M=m, L=l, C=c\right) Pr\left(L=l|X=0, C=c\right)\} Pr\left(M=m|L=l', X=0, C=c\right) Pr\left(L=l'|X=0, C=c\right) \right\} Pr\left(C=c\right).$$

For continuous C, M and L the expression above becomes:

$$\int_c \left\{ \int_{l'} \int_m \left\{ \int_l E\left(Y|X=1, M=m, L=l, C=c\right) f_L\left(l|X=1, C=c\right) - E\left(Y|X=0, M=m, L=l, C=c\right) f_L\left(l|X=0, C=c\right) dl \right\} f_M\left(m|L=l', X=0, C=c\right) f_L\left(l'|X=0, C=c\right) dm\, dl' \right\} f_C\left(c\right) dc.$$

Under the same assumptions, the *TNIE* is identified, for discrete C, M and L, by:

$$\sum_c \left\{ \sum_{l',m,l} E\left(Y|X=1, M=m, L=l, C=c\right) Pr\left(L=l|X=1, C=c\right) \{Pr\left(M=m|X=1, L=l', C=c\right) Pr\left(L=l'|X=1, C=c\right) - Pr\left(M=m|X=0, L=l', C=c\right) Pr\left(L=l'|X=0, C=c\right)\} \right\} Pr\left(C=c\right).$$

For continuous C, M and L the expression above becomes:

$$\int_c \left\{ \int_{l'} \int_m \int_l E\left(Y|X=1, M=m, L=l, C=c\right) f_L\left(l|X=1, C=c\right) \{f_M\left(m|X=1, L=l', C=c\right) f_L\left(l'|X=1, C=c\right) - f_M\left(m|X=0, L=l', C=c\right) f_L\left(l'|X=0, C=c\right)\}\, dl\, dm\, dl' \right\} f_C\left(c\right) dc.$$

Part C: L–Y confounding

Consider the setting where there is an additional variable U that is a common cause of L and Y with the usual assumption of uncorrelated errors. Also assume for simplicity that there are no background confounders C, no interactions, and that all variables have zero mean and unit variance:

$$\begin{cases} Y &= \beta_x X + \beta_l L + \beta_m M + \beta_u U + \epsilon_y \\ M &= \alpha_x X + \alpha_l L + \epsilon_m \\ L &= \gamma_x X + \gamma_u U + \epsilon_l \\ U &= \delta_x X + \epsilon_u \\ X &= \epsilon_x \end{cases} \quad (1)$$

where for completeness we have added an equation for X.

We are interested in what would happen if we omitted U from these models, *i.e.* if we wrongly assumed the model to be:

$$\left\{\begin{array}{lcl} Y & = & \beta'_x X + \beta'_l L + \beta'_m M + \eta_y \\ M & = & \alpha'_x X + \alpha'_l L + \eta_m \\ L & = & \gamma'_x X + \eta_l \\ X & = & \eta_x . \end{array}\right. \tag{2}$$

In this web appendix, we use the theory described by Wermuth and Cox (1) to express the parameters of model (2) in terms of those of model (1). Even though they do not all coincide, we will show that when combined into the causal mediation estimands $CDE(m)$, $PNDE$ and $TNIE$, the bias is compensated, so that both models, (1) and (2), lead to identical mediation estimands.

Model (1) can be re-written as:

$$\begin{array}{lcl} Y - \beta_x X - \beta_l L - \beta_m M - \beta_u U & = & \epsilon_y \\ M - \alpha_x X - \alpha_l L & = & \epsilon_m \\ L - \gamma_x X - \gamma_u U & = & \epsilon_l \\ U - \delta_x X & = & \epsilon_u \\ X & = & \epsilon_x . \end{array}$$

Or equivalently in matrix form

$$\mathbf{A}\mathbf{Y} = \boldsymbol{\epsilon} \tag{3}$$

where $\mathbf{Y} = (Y, M, L, U, X)^T$, $\boldsymbol{\epsilon} = (\epsilon_y, \epsilon_m, \epsilon_l, \epsilon_u, \epsilon_x)^T$, and $\boldsymbol{\Sigma}$, a diagonal matrix, is the variance-covariance matrix of $\boldsymbol{\epsilon}$. $\mathbf{A}$ is then given by:

$$\mathbf{A} = \begin{pmatrix} 1 & -\beta_m & -\beta_l & -\beta_u & -\beta_x \\ 0 & 1 & -\alpha_l & 0 & -\alpha_x \\ 0 & 0 & 1 & -\gamma_u & -\gamma_x \\ 0 & 0 & 0 & 1 & -\delta_x \\ 0 & 0 & 0 & 0 & 1 \end{pmatrix}.$$

After marginalizing over U, our equations are written in matrix form as:

$$\mathbf{A}'\mathbf{Y}' = \boldsymbol{\eta}$$

where $\mathbf{Y}' = (Y, M, L, X)^T$, $\boldsymbol{\eta} = (\eta_y, \eta_m, \eta_l, \eta_x)^T$, and $\mathbf{K}$ is the variance-covariance matrix of $\boldsymbol{\eta}$. $\mathbf{A}'$ is given by:

$$\mathbf{A}' = \begin{pmatrix} 1 & -\beta'_m & -\beta'_l & -\beta'_x \\ 0 & 1 & -\alpha'_l & -\alpha'_x \\ 0 & 0 & 1 & -\gamma'_x \\ 0 & 0 & 0 & 1 \end{pmatrix}.$$

Re-arranging the rows and columns of $\mathbf{A}$ so that U appears first, we re-write equation 3 as

$$\tilde{\mathbf{A}}\tilde{\mathbf{Y}} = \tilde{\boldsymbol{\epsilon}}$$

where

$$\tilde{\mathbf{A}} = \begin{pmatrix} 1 & 0 & 0 & 0 & -\delta_x \\ -\beta_u & 1 & -\beta_m & -\beta_l & -\beta_x \\ 0 & 0 & 1 & -\alpha_l & -\alpha_x \\ -\gamma_u & 0 & 0 & 1 & -\gamma_x \\ 0 & 0 & 0 & 0 & 1 \end{pmatrix}$$

and $\tilde{\mathbf{Y}} = (U, Y, M, L, X)^T$, $\tilde{\boldsymbol{\epsilon}} = (\epsilon_u, \epsilon_y, \epsilon_m, \epsilon_l, \epsilon_x)^T$.

The first step in Wermuth and Cox is to form a matrix $\mathbf{B}$ by partial inversion of $\tilde{\mathbf{A}}$ wrt U.

$$\begin{aligned}\mathbf{B} &= \text{inv}_U \tilde{\mathbf{A}} \\ &= \begin{pmatrix} \tilde{\mathbf{A}}_{UU}^{-1} & -\tilde{\mathbf{A}}_{UU}^{-1}\tilde{\mathbf{A}}_{U\bar{U}} \\ \tilde{\mathbf{A}}_{\bar{U}U}\tilde{\mathbf{A}}_{UU}^{-1} & \tilde{\mathbf{A}}_{\bar{U}\bar{U}} - \tilde{\mathbf{A}}_{\bar{U}U}\tilde{\mathbf{A}}_{UU}^{-1}\tilde{\mathbf{A}}_{U\bar{U}} \end{pmatrix}\end{aligned} \tag{4}$$

where

$$\tilde{\mathbf{A}}_{\bar{U}\bar{U}} = \begin{pmatrix} 1 & -\beta_m & -\beta_l & -\beta_x \\ 0 & 1 & -\alpha_l & -\alpha_x \\ 0 & 0 & 1 & -\gamma_x \\ 0 & 0 & 0 & 1 \end{pmatrix},$$

$$\tilde{\mathbf{A}}_{U\bar{U}} = \begin{pmatrix} 0 & 0 & 0 & -\delta_x \end{pmatrix},$$

$$\tilde{\mathbf{A}}_{\bar{U}U} = \begin{pmatrix} -\beta_u \\ 0 \\ -\gamma_u \\ 0 \end{pmatrix}$$

and

$$\tilde{\mathbf{A}}_{UU} = 1,$$

so that $\tilde{\mathbf{A}}$ partitions as

$$\tilde{\mathbf{A}} = \left(\begin{array}{c|c} \tilde{\mathbf{A}}_{UU} & \tilde{\mathbf{A}}_{U\bar{U}} \\ \hline \tilde{\mathbf{A}}_{\bar{U}U} & \tilde{\mathbf{A}}_{\bar{U}\bar{U}} \end{array}\right).$$

Here $\bar{U}$ refers to "everything but U", and we use this notation for partitioning matrices throughout.

Evaluating equation 4, we obtain:

$$\mathbf{B} = \begin{pmatrix} 1 & 0 & 0 & 0 & \delta_x \\ -\beta_u & 1 & -\beta_m & -\beta_l & -(\beta_x + \beta_u\delta_x) \\ 0 & 0 & 1 & -\alpha_l & -\alpha_x \\ -\gamma_u & 0 & 0 & 1 & -(\gamma_x + \gamma_u\delta_x) \\ 0 & 0 & 0 & 0 & 1 \end{pmatrix}.$$

Let

$$\mathbf{B}_{\bar{U}\bar{U}} = \begin{pmatrix} 1 & -\beta_m & -\beta_l & -(\beta_x + \beta_u\delta_x) \\ 0 & 1 & -\alpha_l & -\alpha_x \\ 0 & 0 & 1 & -(\gamma_x + \gamma_u\delta_x) \\ 0 & 0 & 0 & 1 \end{pmatrix}.$$

Next, since we wish to know the coefficients of the regression of Y on M, L and X when U is ignored, we form the matrix $\mathbf{C}$, which is the partial inversion of $\tilde{\mathbf{B}}_{\bar{U}\bar{U}}$ wrt Y. Applying the partial inversion formula:

$$\mathbf{C} = \begin{pmatrix} 1 & \beta_m & \beta_l & (\beta_x + \beta_u\delta_x) \\ 0 & 1 & -\alpha_l & -\alpha_x \\ 0 & 0 & 1 & -(\gamma_x + \gamma_u\delta_x) \\ 0 & 0 & 0 & 1 \end{pmatrix}.$$

Wermuth and Cox (1) show that the coefficients of the regression of Y on M, L and X when U is ignored are given by:

$$\mathbf{C}_{Y,\bar{Y}} + \mathbf{C}_{Y,Y}\mathbf{Q}_{Y,\bar{Y}}\mathbf{C}_{\bar{Y},\bar{Y}} \tag{5}$$

where

$$\mathbf{Q} = \text{inv}_{\bar{Y}}\tilde{\mathbf{W}}$$

and $\mathbf{W}$ is the variance-covariance matrix of the vector

$$\left(\eta_Y, (\eta_M, \eta_L, \eta_X) - \left(\mathbf{C}_{\bar{Y},Y}\eta_Y\right)^T\right)^T$$

and $\tilde{\mathbf{W}}$ is this matrix rearranged so that the row and column corresponding to Y appears last (so that the order of the variables is M, L, X, Y).

In our setting, $C_{\bar{Y},Y} = (0,0,0)^T$ and so $\mathbf{W}$ is equal to $\mathbf{K}$, the variance-covariance matrix of $\boldsymbol{\eta}$. Wermuth and Cox show that this can be derived as

$$\mathbf{K} = \mathbf{\Sigma}_{\bar{U}\bar{U}} + \mathbf{B}_{\bar{U}U}\mathbf{\Sigma}_{UU}\mathbf{B}_{\bar{U}U}^T,$$

which gives

$$\mathbf{K} = \begin{pmatrix} \sigma_y^2 + \beta_u^2\sigma_u^2 & 0 & \beta_u\gamma_u\sigma_u^2 & 0 \\ 0 & \sigma_m^2 & 0 & 0 \\ \beta_u\gamma_u\sigma_u^2 & 0 & \sigma_l^2 + \gamma_u^2\sigma_u^2 & 0 \\ 0 & 0 & 0 & \sigma_x^2 \end{pmatrix}.$$

Thus

$$\tilde{\mathbf{W}} = \begin{pmatrix} \sigma_m^2 & 0 & 0 & 0 \\ 0 & \sigma_l^2 + \gamma_u^2\sigma_u^2 & 0 & \beta_u\gamma_u\sigma_u^2 \\ 0 & 0 & \sigma_x^2 & 0 \\ 0 & \beta_u\gamma_u\sigma_u^2 & 0 & \sigma_y^2 + \beta_u^2\sigma_u^2 \end{pmatrix}$$

and

$$\tilde{\mathbf{Q}} = \begin{pmatrix} \sigma_m^{-2} & 0 & 0 & 0 \\ 0 & \left(\sigma_l^2 + \gamma_u^2\sigma_u^2\right)^{-1} & 0 & \beta_u\gamma_u\sigma_u^2\left(\sigma_l^2 + \gamma_u^2\sigma_u^2\right)^{-1} \\ 0 & 0 & \sigma_x^{-2} & 0 \\ 0 & \beta_u\gamma_u\sigma_u^2\left(\sigma_l^2 + \gamma_u^2\sigma_u^2\right)^{-1} & 0 & \sigma_y^2 + \beta_u^2\sigma_u^2 - \left(\beta_u\gamma_u\sigma_u^2\right)^2\left(\sigma_l^2 + \gamma_u^2\sigma_u^2\right)^{-1} \end{pmatrix}.$$

Returning to expression (5), we obtain that the coefficients of the regression of Y on M, L and X when U is ignored are given by:

$$\begin{aligned} &\mathbf{C}_{Y,\bar{Y}} + \mathbf{C}_{Y,Y}\mathbf{Q}_{Y,\bar{Y}}\mathbf{C}_{\bar{Y},\bar{Y}} \\ &\quad = (\beta_m, \beta_l, \beta_x + \beta_u\delta_x) + \left(0, \left(\beta_u\gamma_u\sigma_u^2\right)\left(\sigma_l^2 + \gamma_u^2\sigma_u^2\right)^{-1}, 0\right)\begin{pmatrix} 1 & -\alpha_l & -\alpha_x \\ 0 & 1 & -\left(\gamma_x + \gamma_u\delta_x\right) \\ 0 & 0 & 1 \end{pmatrix} \\ &\quad = \left(\beta_m, \beta_l + \beta_u\gamma_u\sigma_u^2\left(\sigma_l^2 + \gamma_u^2\sigma_u^2\right)^{-1}, \beta_x + \beta_u\delta_x - \left(\gamma_x + \gamma_u\delta_x\right)\beta_u\gamma_u\sigma_u^2\left(\sigma_l^2 + \gamma_u^2\sigma_u^2\right)^{-1}\right). \end{aligned} \tag{6}$$

It remains to write σ_l^2 and σ_u^2 in terms of the elements of $\mathbf{A}$.

$$\begin{aligned} \mathrm{Var}\left(U\right) &= \mathrm{Var}\left(\delta_x X + \epsilon_u\right) \\ &= \delta_x^2\mathrm{Var}\left(X\right) + 2\delta_x\mathrm{Cov}\left(X, \epsilon_u\right) + \mathrm{Var}\left(\epsilon_u\right) \\ \Rightarrow 1 &= \delta_x^2 + \mathrm{Var}\left(\epsilon_u\right) \end{aligned}$$

since $\mathrm{Var}\left(U\right) = \mathrm{Var}\left(X\right) = 1$ and $\mathrm{Cov}\left(X, \epsilon_u\right) = 0$ by the uncorrelated errors assumption.

This gives us

$$\mathrm{Var}\left(\epsilon_u\right) = 1 - \delta_x^2.$$

Then, for σ_l^2:

$$\begin{aligned}
\text{Var}\left(L\right) &= \text{Var}\left(\gamma_x X + \gamma_u U + \epsilon_l\right) \\
&= \text{Var}\left(\gamma_x X + \gamma_u U\right) + 2\text{Cov}\left(\gamma_x X + \gamma_u U, \epsilon_l\right) + \text{Var}\left(\epsilon_l\right) \\
&= \text{Var}\left(\gamma_x X + \gamma_u U\right) + \text{Var}\left(\epsilon_l\right) \;\; \text{(uncorrelated errors)} \\
&= \gamma_x^2 \text{Var}\left(X\right) + 2\gamma_x\gamma_u \text{Cov}\left(X, U\right) + \gamma_u^2 \text{Var}\left(U\right) + \text{Var}\left(\epsilon_l\right) \\
&= \gamma_x^2 + 2\gamma_x\gamma_u \text{Cov}\left(X, U\right) + \gamma_u^2 + \text{Var}\left(\epsilon_l\right) \;\; \text{(due to standardisation)} \\
&= \gamma_x^2 + 2\gamma_x\gamma_u \text{Cov}\left(X, \delta_x X + \epsilon_u\right) + \gamma_u^2 + \text{Var}\left(\epsilon_l\right) \\
&= \gamma_x^2 + 2\gamma_x\gamma_u\delta_x + \gamma_u^2 + \text{Var}\left(\epsilon_l\right) \\
\Rightarrow 1 &= \gamma_x^2 + 2\gamma_x\gamma_u\delta_x + \gamma_u^2 + \text{Var}\left(\epsilon_l\right).
\end{aligned}$$

This gives us

$$\text{Var}\left(\epsilon_l\right) = 1 - \gamma_x^2 - 2\gamma_x\gamma_u\delta_x - \gamma_u^2.$$

Putting these back into equation (6), we obtain:

$$\begin{aligned}
\beta_m' &= \beta_m \\
\beta_l' &= \beta_l + \frac{\beta_u\gamma_u\left(1-\delta_x^2\right)}{1 - \gamma_x^2 - 2\gamma_x\gamma_u\delta_x - \gamma_u^2 + \gamma_u^2\left(1-\delta_x^2\right)} \\
&= \beta_l + \frac{\beta_u\gamma_u\left(1-\delta_x^2\right)}{1 - \left(\gamma_x + \gamma_u\delta_x\right)^2} \\
\beta_x' &= \beta_x + \beta_u\delta_x - \frac{\left(\gamma_x + \gamma_u\delta_x\right)\beta_u\gamma_u\left(1-\delta_x^2\right)}{1 - \gamma_x^2 - 2\gamma_x\gamma_u\delta_x - \gamma_u^2 + \gamma_u^2\left(1-\delta_x^2\right)} \\
&= \beta_x + \beta_u\delta_x - \frac{\left(\gamma_x + \gamma_u\delta_x\right)\beta_u\gamma_u\left(1-\delta_x^2\right)}{1 - \left(\gamma_x + \gamma_u\delta_x\right)^2}.
\end{aligned}$$

Going through the same calculations for the regression coefficients for L and X in the equation for M (after marginalising over U), and for the regression coefficient for X in the equation for L (after marginalising over U), we similarly obtain:

$$\begin{aligned}
\alpha_l' &= \alpha_l \\
\alpha_x' &= \alpha_x \\
\gamma_x' &= \gamma_x + \delta_x\gamma_u.
\end{aligned}$$

These results are intuitive, since U does not appear in the true data generating equation for M, and since marginalising over U apportions the path from X to U to L to the path from X to L.

Putting all this into the expressions for the *PNDE*, *TNIE* and *CDE*, we obtain:

$$\begin{aligned}
\mathit{CDE}(m)' = \mathit{PNDE}' &= \beta_x' + \gamma_x'\beta_l' \\
&= \beta_x + \beta_u\delta_x - \frac{\left(\gamma_x + \gamma_u\delta_x\right)\beta_u\gamma_u\left(1-\delta_x^2\right)}{1 - \left(\gamma_x + \gamma_u\delta_x\right)^2} + \left(\gamma_x + \delta_x\gamma_u\right)\left\{\beta_l + \frac{\beta_u\gamma_u\left(1-\delta_x^2\right)}{1 - \left(\gamma_x + \gamma_u\delta_x\right)^2}\right\} \\
&= \beta_x + \beta_u\delta_x + \beta_l\left(\gamma_x + \delta_x\gamma_u\right) \\
&= \mathit{PNDE} = \mathit{CDE}(m)
\end{aligned}$$

and

$$\begin{aligned}
\mathit{TNIE}' &= \beta_m'\left(\alpha_x' + \gamma_x'\alpha_l'\right) \\
&= \beta_m\left\{\alpha_x + \left(\gamma_x + \gamma_u\delta_x\right)\alpha_l\right\} \\
&= \mathit{TNIE}.
\end{aligned}$$

Thus, when a (background or intermediate) confounder U of the L–Y relationship is ignored, even though some of the individual SEM parameters are biased (*i.e.* cannot be given a causal interpretation as data generating parameters), this bias is compensated when the mediation estimands are calculated, so that the *PNDE*, *TNIE*, and *CDE* can be identified without data on U.

WEB TABLE 1

Estimated coefficients of the SEMs corresponding to the models reported in Table 3; ALSPAC Study; United Kingdom 1991-2005; N=2,749.

Outcome variable		Explanatory variable		Parameter	Model 1[a] Estimate	Model 1[a] SE	Model 1[a] p-value	Model 2[a] Estimate	Model 2[a] SE	Model 2[a] p-value
Y	'Bingeing/Overeating'									
		X	High maternal BMI	β_x	0.072	0.048	0.14	0.094	0.049	0.05
		M	Childhood BMI score	β_m	0.315	0.019	<0.001	0.315	0.021	<0.001
		M^2	(Childhood BMI score)^2	β_{mm}	0.044	0.012	<0.001	0.043	0.012	<0.001
		L	Birth weight score	β_l	0.034	0.022	0.13	0.049	0.019	0.01
		L^2	(Birth weight score)^2	β_{ll}	0.032	0.012	0.01	-	-	-
		XL	High mat BMI × BW	β_{xl}	0.079	0.045	0.08	-	-	-
		XM	High mat BMI × ch BMI	β_{xm}	-	-	-	0.018	0.045	0.69
		C_1	Low maternal education	β_{c1}	-0.011	0.036	0.76	-0.011	0.036	0.76
		C_2	Poor maternal mental health	β_{c2}	0.207	0.054	<0.001	0.211	0.054	<0.001
M	Childhood BMI score									
		X	High maternal BMI	α_x	0.544	0.048	<0.001	0.544	0.048	<0.001
		L	Birth weight score	α_l	-0.025	0.023	0.28	-0.025	0.023	0.28
		XL	High mat BMI × BW	α_{xl}	0.073	0.046	0.11	0.073	0.046	0.11
		C_1	Low maternal education	α_{c1}	0.156	0.037	<0.001	0.156	0.037	<0.001
		C_2	Poor maternal mental health	α_{c2}	0.022	0.056	0.69	0.022	0.056	0.69
L	Birth weight score									
		X	High maternal BMI	γ_x	0.223	0.045	<0.001	0.223	0.045	<0.001
		C_1	Low maternal education	γ_{c1}	-0.065	0.035	0.07	-0.065	0.035	0.07
		C_2	Poor maternal mental health	γ_{c2}	-0.054	0.053	0.31	-0.054	0.053	0.31

♯ BMI: body mass index; SE: standard error; BW: birth weight.
[a] Model 1 assumes the Robins and Greenland assumption that there is no X–M interaction at the individual level in their effects on Y (2).
[b] Model 2 assumes the Petersen *et al.* assumption that (conditional on C) the controlled direct effect does not vary with $M(0)$ (3).

REFERENCES

1. Wermuth N, Cox DR. Distortion of effects caused by indirect confounding. *Biometrika.* 2008;95(1):17–33.

2. Robins JM, Greenland S. Identifiability and exchangeability for direct and indirect effects. *Epidemiology.* 1992;3(2):143–155.

3. Petersen ML, Sinisi SE, van der Laan MJ. Estimation of direct causal effects. *Epidemiology.* 2006;17(3):276–284.
